# Thermal Insulation and Compressive Performances of 3D Printing Flexible Load-Bearing and Thermal Insulation Integrated Lattice

**DOI:** 10.3390/ma15238625

**Published:** 2022-12-02

**Authors:** Xin Wang, Ang Li, Xuefeng Liu, Xiangrui Wan

**Affiliations:** 1 Beijing Advanced Innovation Center for Materials Genome Engineering, University of Science and Technology Beijing, Beijing 100083, China; 2 Beijing Laboratory of Metallic Materials and Processing for Modern Transportation, University of Science and Technology Beijing, Beijing 100083, China; 3 Key Laboratory for Advanced Materials Processing of Ministry of Education, University of Science and Technology Beijing, Beijing 100083, China; 4 School of Materials Science and Engineering, Tsinghua University, Beijing 100080, China

**Keywords:** additive manufacturing, SLM, lattice structures, thermal insulation performance, compressive performance

## Abstract

Structurally and functionally integrated materials usually face the problem of serious functional degradation after large deformation or fracture, such as load-bearing and thermal insulation integrated lattice. In this work, the lattice with a big width-thickness ratio, which empowered the flexibility of the lattice by reducing the rod deformation during compression, was proposed. The structure of the lattice almost kept integrality after large deformation or fracture, and the decay of thermal insulation performance was less. Compared with the conventional lattice, the big width-thickness ratio lattice obtained favorable thermal insulation performance. On this basis, two kinds of flexible load-bearing and thermal insulation integrated hourglass lattices with big width-thickness ratios (BWR lattice) were prepared by SLM, and the thermal insulation and compressive performances were measured. The thermal insulation efficiency could reach 83% at 700 °C. The lattice would recover after large deformation or fracture, and the thermal insulation efficiency of the fracture lattice was 75%. This work provides a new way of designing load-bearing and thermal insulation integrated lattice and achieves the functionality preservation of load-bearing and thermal insulation integrated lattice after large deformations and fractures.

## 1. Introduction

With the development of the aerospace industry, aircrafts face more and more extreme external environments. New challenges to the thermal insulation and load-bearing performance of the aircraft were posed by the complex temperature and mechanical load variations [1,2,3]. Common load-bearing and thermal insulation integrated structures include metal foams, sandwich structures, metal lattices, etc. [4,5,6,7]. Among them, metal lattices obtain the advantages of lightweight, high specific strength, and buffered energy absorption [8,9,10], and attract the interest of more and more scholars. However, when encountering sudden situations such as collision and extrusion, the aircraft is prone to incur large deformation, which results in the buckling and fracture of the rods [11]. The lattices are compacted, and thermal short circuits occur [12]. This problem of severe degradation of thermal insulation performance when the lattice is destroyed seriously affects the flight safety of the aircraft.

To solve this problem, scholars have carried out relevant research work, which is currently focused on improving the stiffness of the lattice and suppressing structural deformation by adding support structures [13,14]. It usually leads to a mass increase. In addition, the existing metal lattice has limitations in thermal insulation performance and is used with additional thermal insulation materials such as ceramic fiber and aerogel [15]. It increases the manufacturing cost and process complexity, and the additional thermal insulation materials are susceptible to fracture during deformation, which is the important reason for the decline in thermal insulation performance.

Improving the flexibility of the lattice could effectively inhibit the destructive fracture occurring under the condition of large deformation, and the common methods are using flexible materials and designing flexible structures [16,17,18]. Some scholars have prepared flexible ceramic aerogel material to solve the problem of serious thermal insulation performance degradation which is caused by the destruction of rigid ceramic aerogel material after thermal shock [19]. However, the flexibility of metals is too low to meet the requirement, hence the structural design is required.

The main reason for compressive failure in the existing metal lattice is the deformation along the rod direction exceeding the material yield limitation [20]. By reducing the rotation angle of the rod in the compressive direction during loading, the deformation along the rod could be effectively reduced. If the rod deformation is always less than the material yield limit, the lattice will not be a destructive fracture and will recover after load release. It shows the flexibility along the thickness direction. By increasing the width-thickness ratio of the core in the lattice, the angle of rod rotation during compression can be effectively reduced, thus improving the flexibility of the dot matrix.

In addition, increasing the width-thickness ratio could also improve the thermal insulation performance of the lattice. Generally, thermal conduction is the most important thermal transfer way of metal lattice [21,22]. According to Fourier’s law [23], the thermal flow generated by thermal conduction is inversely proportional to the transfer distance. Increasing the length of the rod in a single core can extend the thermal transfer distance and reduce the thermal transfer while keeping thickness constant.

In summary, this work proposes a flexible metal lattice that mainly relies on the structure to achieve high thermal insulation performance. It solves the problem of serious thermal insulation performance degradation after the destruction of the lattice. In this work, two kinds of flexible load-bearing and thermal insulation integrated hourglass lattice with a big width-thickness ratio (hereinafter referred to as “BWR lattice”) were designed by considering the deformation and thermal transfer capability of TC4, and then they were manufactured by SLM. The thermal insulation and compressive performances of the BWR lattices were studied. The thermal transfer model was established, and the thermal transfer process was analyzed. The resilient and cycle resilient performances of the lattice were also discussed. This work provides a new way of designing load-bearing and thermal insulation integrated lattice and achieves the functionality preservation of load-bearing and thermal insulation integrated lattice after large deformations and fractures. It is expected to promote further development and application in the field of thermal protection.

## 2. Materials and Methods

### 2.1. BWR Lattice Structures

**Figure 1:**
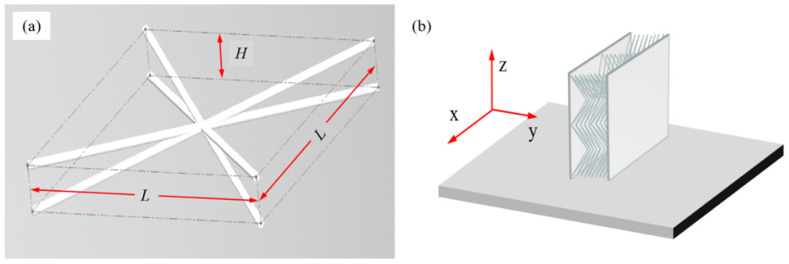
Design and 3D printing of the BWR lattice. (**a**) Core of the BWR lattice; (**b**) Vertical placement for specimen.

As shown in Figure 1, the width and thickness of the BRW lattice were L/mm and H/mm, respectively. The width-thickness ratio k was equal to L/H. The specimens with the k of 6 and 8 were printed (S-1 and S-2) because larger or lower k would lead to poor mechanical performance or thermal insulation performance. To facilitate the measurements, panels with a thickness of 2 mm were added at both the top and bottom of the BWR lattice, and other dimensions were shown in Table 1.

### 2.2. Materials

The TC4 powders were provided by Avimetal Powder Metallurgy Technology (Beijing, China) Co., Ltd. The particle size of the powder was 15–53 μm and the components were shown in Table 2. Butane was used for the combustion experiments in this work.

### 2.3. Additive Manufacturing Process

The SLM printer (SLM100) was provided by Guangdong Hanbang 3D Tech Co., Ltd (Zhongshan, China). The BWR lattice was performed using a vertical placement as shown in Figure 1b, owing to the short projection of the core rod in the XY plane. It had good self-supporting properties and therefore improved the quality of the printing. The process parameters were shown in Table 3.

### 2.4. Characterizations

**Figure 2:**
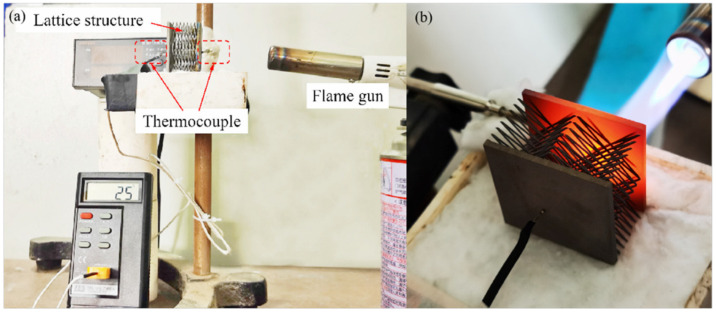
Measurement of thermal insulation performance for the BWR lattice: (**a**) Image of thermal insulation performance test platform; (**b**) Positions of thermocouples.

As shown in Figure 2a, the test platform was built and the thermal insulation performance of the BWR lattice was measured by a combustion experiment. The lattice was placed vertically on the asbestos felt to minimize bottom contact surface thermal dissipation. Thermocouples were used to measure the real-time temperature of the heated surface (directly exposed to the flame jet) and the insulated surface (insulated by lattice structure). A flame gun was used for heating, and the flame temperature was controlled by adjusting the flame size and the distance between the gun and the heated surface. The time of heating was 10 min. The thermocouple of the heated surface was placed at the center of the flame. In addition, the temperature of the heated surface was maintained above 700 °C. The thermocouple of the insulated surface was facing the center of the flame to eliminate the experimental error introduced by the deviation of the thermocouple position, as shown in Figure 2b.

According to ASTM C365/C365M-2011 standard, a multifunctional mechanical property testing machine was used to measure the compressive performance of the lattice structures at the rate of 2 mm/min.

## 3. Results

### 3.1. Relative Density

Relative density was the key factor for evaluating the lightweight qualification of the lattice. According to the definition, relative density *ρ*_0_ could be calculated by the equation below:

(1) $$\rho_{0}=\frac{\rho_{L}}{\rho_{Ti}}\times 100\%$$

where *ρ_Ti_*/g·mm^−3^ was the density of TC4, *ρ_L_*/g·mm^−3^ was the real density of lattice. According to the two calculation methods for lattice mass *m_L_*/g, *ρ_L_* could be calculated by Equation (3):

(2) $$m_{L}=\rho_{Ti}\cdot V_{L}=\rho_{L}\cdot V_{T}$$

(3) $$\rho_{L}=\rho_{Ti}\cdot \frac{V_{L}}{V_{T}}=\rho_{Ti}\cdot \frac{\pi d^{2}\sqrt{2L^{2}{+H}^{2}}}{L^{2}H}$$

where *d*/mm was the diameter of the core rod, *V_T_*/mm^3^ was the volume of the single core, and *V_L_*/mm^3^ was the real volume of the lattice.

**Figure 3:**
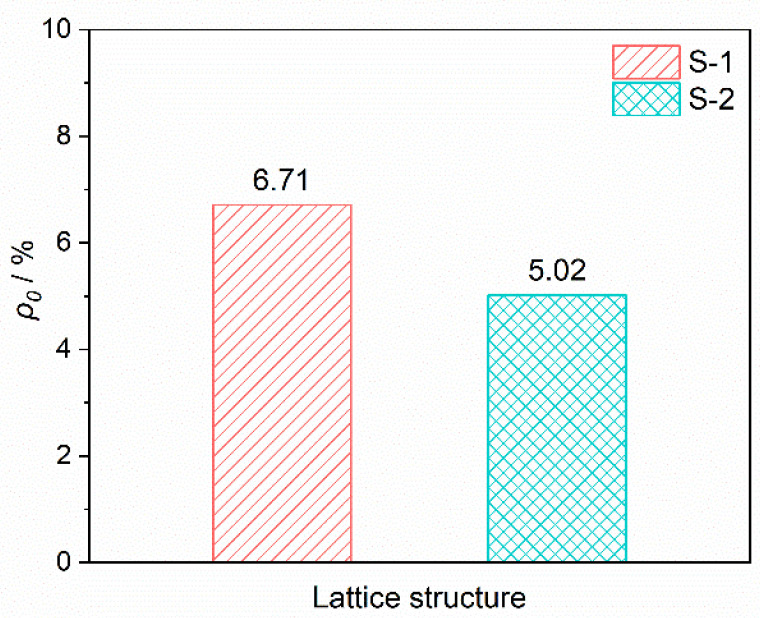
Relative density of the BWR lattices.

As shown in Figure 3, *ρ*_0_ of the BWR lattices were calculated by Equations (1) and (3), and it was obvious that the lightweight level of S-2 was higher. There was less impact for *ρ*_0_ on the mechanical and thermal insulation performances for both the two lattices because of the low values. Hence, the influence taken from the difference of *ρ*_0_ was ignored in this work.

### 3.2. Thermal Insulation Performance

**Figure 4:**
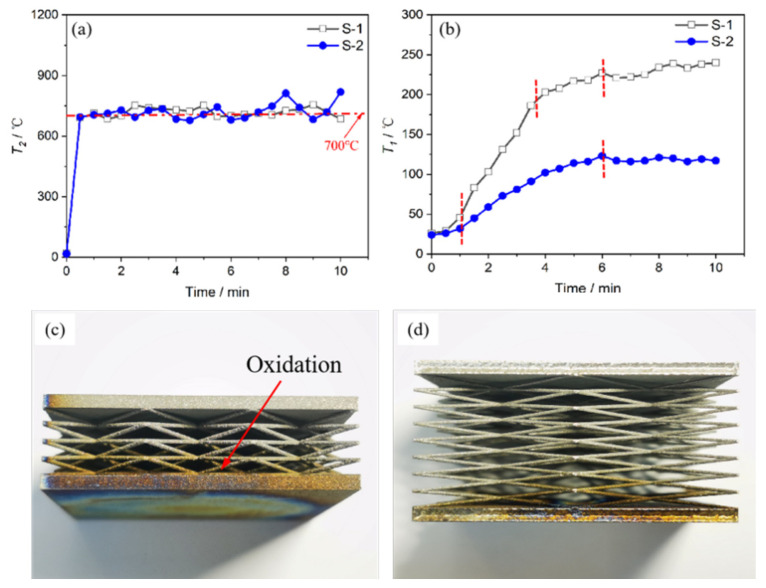
Thermal insulation performance of the BWR lattices: (**a**) Curve of *T*_2_ with heating time; (**b**) Curve of *T*_1_ with heating time; (**c**) Image of S-1 after heating; (**d**) Image of S-2 after heating.

The curve of heated surface temperature (*T*_2_/°C) with heating time was shown in Figure 4a. *T*_2_ would reach and stabilize around the aimed temperature in 30 s, which was 700 °C. To simplify the calculations, *T*_2_ was taken as 700 °C in the subsequent discussions.

The curve of insulated surface temperature (*T*_1_/°C) with heating time was shown in Figure 4b. There were four stages, which were preheated, high speed rising, low speed rising, and stability, in the curve of *T*_1_ for S-1 (*T*_1_^S−1^). *T*_1_^S−1^ reached the maximum (230 °C) at the time of 6 min and then stayed constant. Different from S-1, there were only three stages, which were preheated, low speed rising, and stability, in the curve of *T*_1_ for S-2 (*T*_1_^S−2^). This might be due to the difference in thermal conductivity between S-1 and S-2. This is a topic for further research, as the balance temperature was in the focus of this paper. Generally, the thermal insulation performance could be evaluated by thermal insulation efficiency. It was calculated by dividing the temperature difference between *T*_1_ and *T*_2_ by *T*_2_. At 700 °C, the thermal insulation efficiency of S-2 reached 83% and that of S-1 was 67%. S-2 showed favorable thermal insulation performance.

By comparison with the published data [24], it was found that the thermal insulation performance of S-2 was close to that of Ni honeycomb laminate, which was commonly used in aerospace and achieved a thermal insulation efficiency of 84.6% at 650 °C. It was worth noting that the Ni honeycomb laminate had an additional 25 mm ceramic insulation layer (the total thickness was 32 mm), while S-2 was an all-metal structure with a smaller total thickness.

The images of S-1 and S-2 (after heating) were shown in Figure 4c,d. The oxidation of the rod close to the heated surface was intense and the color was changed. With the increasing distance between the rod and the heated surface, the oxidation was weakened. In addition, it could be observed that the oxidation of some rod connection positions was intense, while that of the adjacent rods was weak (Supplementary Figure S1). It indicated that the oxidative discoloration at the rod connection position was caused by thermal radiation rather than thermal conduction. Therefore, thermal radiation also had an important effect on *T*_1_ in the BWR lattice. This will be discussed in Section 4.1 in detail.

### 3.3. Compressive Performance

**Figure 5:**
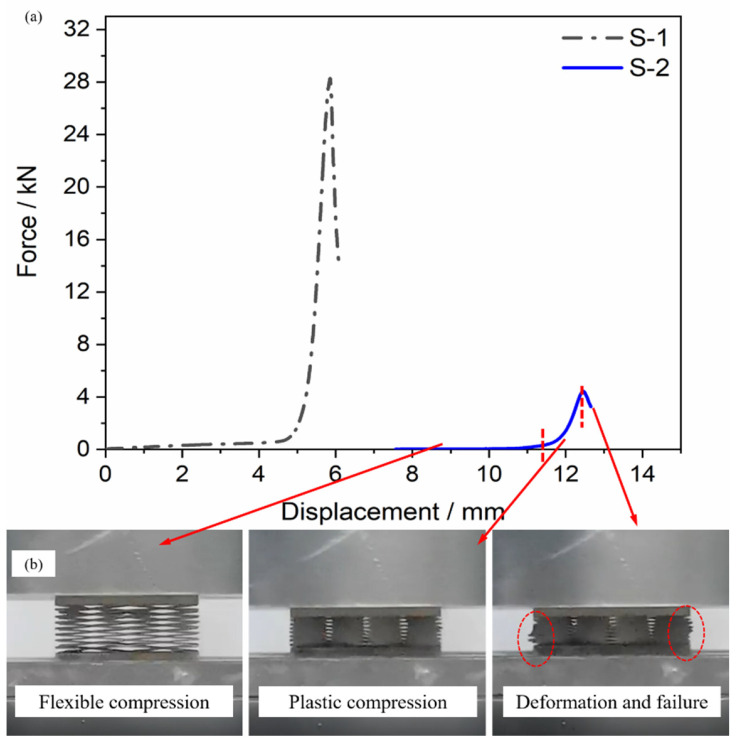
Compressive performance of the BWR lattice: (**a**) Force-Displacement curve of the BWR lattice; (**b**) Compressive process of S-2.

The force–distance curves of the BWR lattices are shown in Figure 5a. The compressive process could be divided into three stages. In the first stage, only elastic deformation occurred, and the curve was approximately a straight line. In the second stage, the lattice deformed plastically, and the slope of the curve increased. The load-bearing capacity of the lattice rose rapidly and reached its extreme value. In the third stage, owing to the compressive failure of the lattice under excessive load, the curve fell rapidly, as shown in Figure 5b.

Different from the traditional rigid lattice, the load-bearing capacity of the flexible lattice should be characterized by not only the ultimate compressive force but also the strength during elastic deformation. The former indicated the maximum load of the lattice in case of large deformation, and the latter indicated the load-bearing capacity of the lattice with the complete thermal insulation capacity. Compared with S-2, S-1 obtained a better ultimate compressive force. It was attributed to the smaller cores of S-1 which were distributed more in the same cross-section. S-2 obtained the greater elastic deformation performance. It was attributed to a bigger width-thickness ratio which could reduce the deformation of the rod connection positions during deformation. In addition, the flexible lattices had a certain load-bearing capacity when only elastic deformation occurred.

All above, S-1 could bear more loads, while S-2 was highly flexible and had more favorable insulation performance. The two lattices could be applied to different application scenarios, respectively.

### 3.4. Cycle Resilient Performance

The resilient rate *η* was used to characterize the recovery properties of the BWR lattice (after compressive failure). It was the ratio of the difference between the lattice recovery thickness *H_r_*/mm and the broken thickness *H_b_*/mm and could be calculated by Equation (4). The deformation and recovery ability of the lattice were both taken into account. Compared with the recovery rate, it provided a more intuitive representation of the lattice flexibility.

(4) $$\eta =\frac{H_{r}-H_{b}}{H_{b}}$$

**Figure 6:**
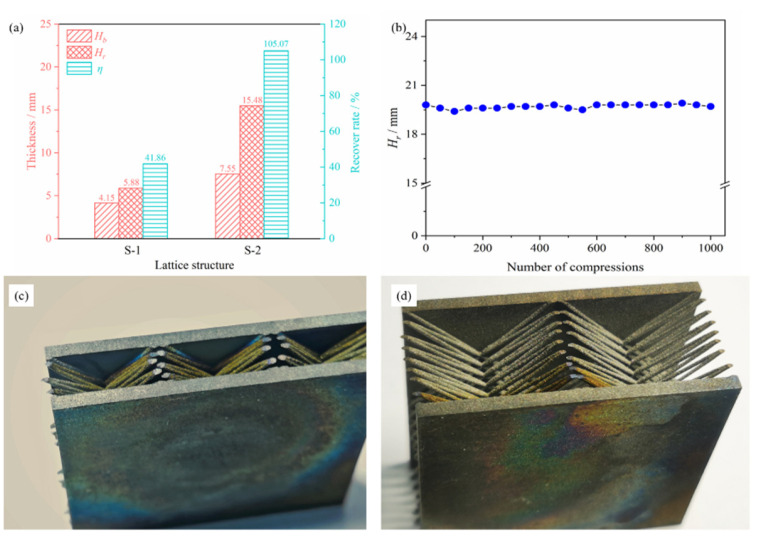
Resilient and cycle resilient performance of the BWR lattices: (**a**) Resilient performance; (**b**) Cycle resilient performance of S-2; (**c**) Image of the recovered S-1; (**d**) Image of the recovered S-2.

The thickness and resilient rate of the BWR lattices were shown in Figure 6a. It was obvious that the recovery properties of S-2 were favorable. The *η* of S-2 reached 105.07%, which was far beyond the *η* of S-1 (41.86%). It indicated that S-2 was still highly flexible after compressive failure. The morphologies of the recovered S-1 and S-2 were shown in Figure 6c,d. The deformation of S-1 was more serious, and the rods showed obvious misalignment, while the deformation of S-2 was smaller, and the lattice basically kept the original structure. The resilient rate was closely related to the lattice structure and the positions of compressive failure, and it would be discussed in Section 4.2 in detail.

To further research the cycle resilient performance of the lattice, the recovery thickness of compressive failure S-2 continued for 0–1000 deformation cycles was tested in this work, and the results were shown in Figure 6b. It was worth noting that the *H_r_* did not decay. The BWR lattice exhibited favorable resilient properties after compressive failure and cyclic deformation, which played a key role in maintaining thermal insulation performance. It would be discussed in Section 4.2 in detail.

## 4. Discussions

### 4.1. Thermal Transfer Process of the BWR Lattice

#### 4.1.1. Calculations of Thermal Flow and Equivalent Coefficient of Thermal Conductivity

Generally, there are three ways of thermal transfer: thermal conduction, heat radiation, and heat convection. Since the air inside the BWR lattices hardly flowed, the thermal convection was not significant [25]. Only the influences of thermal conduction and thermal radiation (from the panels) on the thermal transfer process of the lattice were considered in this work. The thermal flow from thermal conduction and thermal radiation would be calculated below.

First, the thermal flow generated by thermal conduction *Φ*_0_/W was calculated. Since the thermal transfer process achieved a steady state after the temperature reached equilibrium, according to Fourier’s law [23] (Equation (5)), *Φ*_0_ was calculated.

(5) $$\Phi_{0}{=A}_{0}\lambda_{0}\frac{T_{2}-T_{1}}{S}$$

where *A*_0_/mm^2^ was the area of thermal conduction, *λ*_0_/W·m^−1^·°C^−1^ was the coefficient of thermal conductivity (CTC), and *S*/mm was the distance of thermal conduction. Based on the geometry of the lattice, *A*_0_ and *S* could be calculated.

(6) $$A_{0}=n\pi d^{2}$$

(7) $$S=m\sqrt{2L^{2}+H^{2}}$$

where *n* was the product of the lattice repetitions in the width and length directions, and *m* was the lattice repetitions in the thickness directions.

In addition, the lattice could be equated to a thick panel. The equivalent thermal flow *Φ*_1_/W was calculated.

(8) $$\Phi_{1}{=A}_{1}\lambda_{r}\frac{T_{2}-T_{1}}{mH}$$

where *A*_1_/mm^2^ was the cross-section of the equivalent panel, *λ_r_*/W·m^−1^·°C^−1^ was the equivalent coefficient of thermal conductivity (ECTC) of the lattice. Since *Φ*_2_ and *Φ*_1_ were equal, the formula for *λ_r_* could be obtained by associating Equation (5) with Equation (8).

(9) $$\lambda_{r}=\frac{\pi d^{2}\lambda_{0}H}{L^{2}\sqrt{2L^{2}+H^{2}}}=\frac{\pi d^{2}\lambda_{0}}{L^{2}\sqrt{2{\frac{L}{H}}^{2}+1}}$$

**Figure 7:**
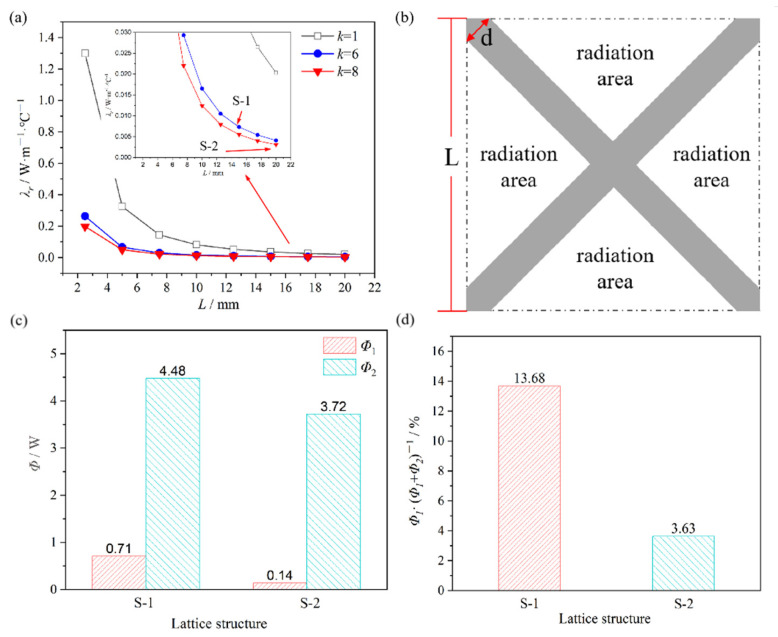
Calculation of lattice thermal conduction performance: (**a**) Equivalent coefficient of thermal conductivity; (**b**) Schematic diagram of thermal radiation; (**c**) Thermal flow of the lattices; (**d**) Ratio of *Φ*1 in thermal flow.

The parameters in Table 4, *T*_0_, and *T*_1_ were brought into Equations (8) and (9) to calculate *λ_r_* and *Φ*_1_ for S-1 and S-2, and the results were shown in Figure 7.

From Equation (9), *λ_r_* was closely related to the *L*, *H*, and *k*, and was independent of *m* and *n*. Hence, the ECTC of the lattice was equal to that of the core. To improve the thermal insulation performance of the lattice, it was necessary to increase the width-thickness ratio as much as possible.

In this work, the ECTC of lattices with different sizes were calculated and the results were shown in Figure 7a. Compared with the common cubic lattice, the BWR lattice obtained a much smaller ECTC when the width was the same.

Secondly, the thermal flow generated by thermal radiation *Φ*_2_/W was calculated, assuming that the radiation occurred only through the panel along the thickness direction and was not generated by the rods. To the thermal radiative transfer model between infinitely large flat panels [23], Equation (10) could be obtained.

(10) $$\Phi_{2}=\frac{\sigma (T_{1}^{4}-T_{2}^{4})}{\frac{1-\varepsilon_{1}}{\varepsilon_{1}A_{f}}+\frac{1}{A_{f}}+\frac{1-\varepsilon_{2}}{\varepsilon_{2}A_{f}}}$$

where *σ* was Stefan-Boltzmann constant 5.67 × 10^−8^ W/(m^2^·°C^4^), *ε*_1_ was the IR emissivity of the heated surface, *ε*_2_ was the IR emissivity of the insulated surface, *A_f_*/mm^2^ was the radiation area of the lattice. The degree of oxidation had a great influence on the IR emissivity of the metal surface. According to the references, *ε*_1_ and *ε*_2_ were taken as 0.8 and 0.2, respectively [26,27]. The formula for *A_f_* was obtained by the geometric relationship of the lattice, as shown in Figure 7b.

(11) $$A_{f}=n{\left(L-\sqrt{2}d\right)}^{2}$$

Hence, *Φ*_2_ of S-1 and S-2 could be calculated by Equations (10) and (11), and the results were shown in Figure 7c. It was noted the influence of lattice on *Φ*_2_ was taken by the blocking for IR. It was consistent with the experimental phenomenon in Section 3.2.

According to the constitutive relationship in the thermal transfer process, the total thermal flow *Φ* of the lattice was equal to the sum of *Φ*_1_ and *Φ*_2_. As shown in Figure 7d, *Φ*_1_ was a relatively low percentage of *Φ*, and thermal radiation was the main way of the thermal transfer for the BWR lattice. It differed from the experience that the main thermal transfer way of the conventional lattice at 700 °C was thermal conduction. The reason was that the thermal conduction of the lattice was suppressed by the big width-thickness ratio structure. It explained why the BWR lattice obtained favorable thermal insulation performance.

#### 4.1.2. Analysis of Thermal Insulation Performance

When the thermal transfer process reached a steady state, the lower the *T*_2_ was, the higher the thermal insulation efficiency. *T*_2_ at a steady state was related to the thermal flux *Φ*′/W·m^−2^ of the insulated surface, hence the thermal insulation performance of the lattice could be improved by decreasing *Φ*′.

According to the definition, *Φ*′ consisted of thermal conduction flux *Φ*_1′_/W and thermal radiation flux *Φ*_2′_/W, and the calculations were performed by the equations below:

(12) $$\Phi_{1}^{\prime }=\frac{\Phi_{1}}{A}=\frac{\pi d^{2}\lambda_{0}\left(T_{1}-T_{2}\right)}{mL^{2}\sqrt{2L^{2}+H^{2}}}$$

(13) $$\Phi_{2}^{\prime }=\frac{\Phi_{2}}{A}={\left(1-\frac{\sqrt{2}}{L}d\right)}^{2}\cdot \frac{\sigma \left(T_{1}^{4}-T_{2}^{4}\right)}{\frac{1-\varepsilon_{1}}{\varepsilon_{1}}+1+\frac{1-\varepsilon_{2}}{\varepsilon_{2}}}$$

(14) $$\Phi^{\prime }=\Phi_{1}^{\prime }+\Phi_{2}^{\prime }$$

**Figure 8:**
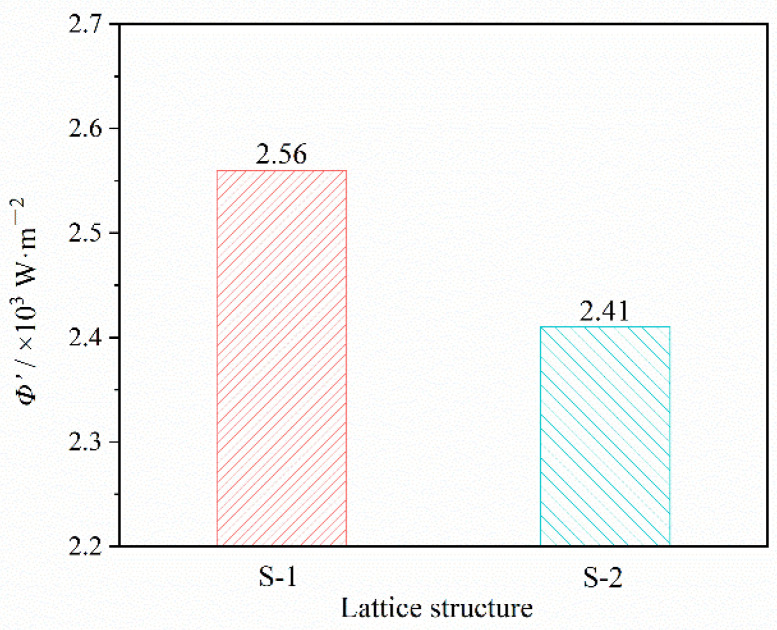
Thermal flux of S-1 and S-2.

It was obvious that *Φ*′ was closely related to the lattice size. The thermal insulation performance could be further improved by structural design. In order to verify the conclusion, the thermal fluxes of S-1 and S-2 were calculated, as shown in Figure 8. The results showed that *Φ*′ of S-2 was smaller and the thermal insulation was better. It was consistent with the experimental result.

In addition, the structure could be redesigned to improve the mechanical properties, lightweight, and manufacturing performance of the lattice, under the condition that *Φ*′ remained unchanged. It is worth noting that the change of lattice structure would lead to the variation of thermal dissipation in practical conditions. Therefore, the variation of thermal dissipation also needed to be considered when redesigning the lattice structure, and the related research was still in progress and will not be discussed in this work.

### 4.2. Resilient Performance of the BWR Lattice

#### 4.2.1. Resilient Mechanism

The BWR lattice proposed in this work could be resilient after large deformation and fracture. It had a favorable cycle resilient performance. It was shown that both the lattice structure and the fracture form would affect the resilient performance by analyzing the press-resilient process.

**Figure 9:**
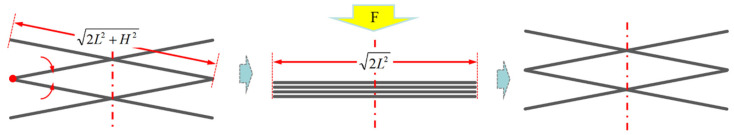
Force analysis of rods.

On the one hand, owing to the big width-thickness ratio, the lattice underwent inward rotation along the rod connection point when it was stressed, and the compressive deformation perpendicular to the cross-section of the rod occurred, as shown in Figure 9. The strain *ε* could be calculated below:

(15) $$\varepsilon =1-\frac{\sqrt{2L^{2}}}{\sqrt{2L^{2}{+H}^{2}}}=1-\frac{1}{\sqrt{1+\frac{1}{2}\cdot {\left(\frac{H}{L}\right)}^{2}}}$$

**Figure 10:**
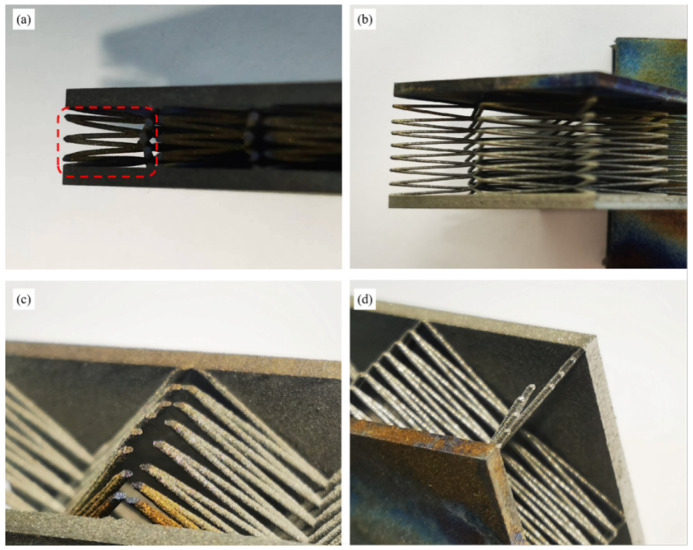
Morphology of lattice after compressive failure: (**a**) Morphology of rods in S-1; (**b**) Morphology of rods in S-2; (**c**) Morphology of rod connection positions in S-2; (**d**) Morphology of rod deformation in S-2.

According to Equation (15), the larger the *k*, the smaller the *ε*. The lattice could recover completely to its original thickness after the load was released when *ε* was less than the elastic deformation limit of the rod. Since the lattice was fractured in the compressive process, the rod underwent not only elastic deformation, but also a certain plastic deformation, and the lattice could not recover completely. Compared with S-1, S-2 obtained the smaller *k*, and the plastic deformation was lighter. As shown in Figure 10a,b, the resilient performance of S-2 was favorable. As the compressive cycle continued, the lattice no longer suffered plastic deformation, so the resilient thickness remained constant.

On the other hand, the fracture of the BWR lattice occurred at the rod connection positions, and only a small amount of bending deformation formed on the rods, as shown in Figure 10c,d. The internal structure of the lattice retained integrity and buckle or fracture did not occur. Hence, the lattice obtained a resilient performance.

#### 4.2.2. Influence of Compressive Failure on Thermal Insulation

**Figure 11:**
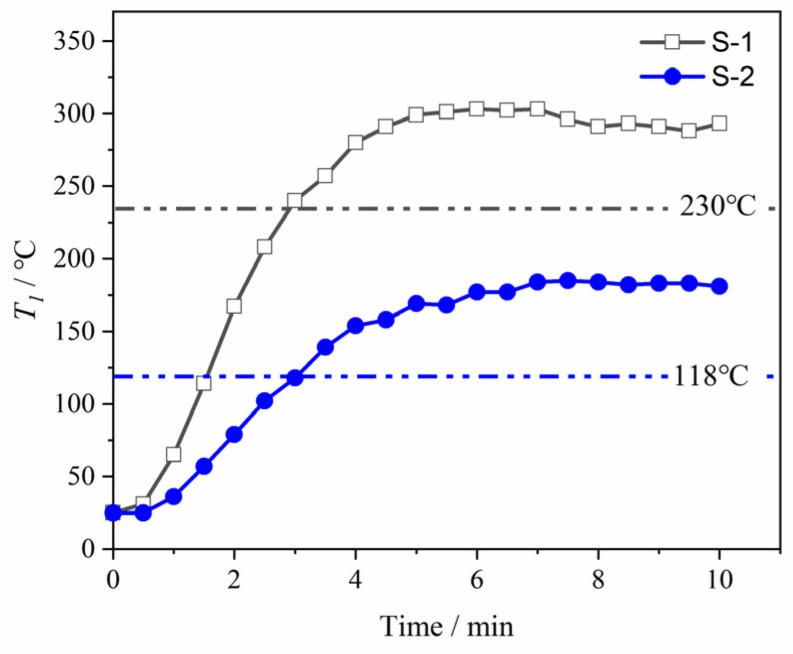
Thermal insulation performance of the lattices after compressive failure.

Partial deformations, which were caused by compressive failure, would affect the thermal insulation performance. The thermal insulation performances of S-1 and S-2 after compressive failure and resilience were measured, as shown in Figure 11. The results showed that the thermal insulation efficiency of S-1 and S-2 were 58% and 75%, respectively. Although the thermal insulation performance of the fracture lattice decayed slightly (8–9%) compared with that of the original lattice, the thermal insulation performance of the fracture lattice still remains favorable.

The reasons for the thermal insulation performance decay of the fracture lattice were analyzed. It was found that the plastic deformation caused by compressive failure increased the contact area between adjacent rods and shortened the path of thermal conduction, which would lead to an increase in thermal flow, as shown in Figure 10a. In addition, the reduction of thermal dissipation caused by the deformation might be a reason for the decay of the thermal insulation performance.

## 5. Conclusions

This work shows that the BWR lattices obtain favorable flexibility and thermal insulation performance. The thermal insulation efficiency of the BWR lattice reaches 83% with any additional thermal insulation materials. It is basically the same as the thermal insulation performance of Ni honeycomb laminate with an additional 25 mm ceramic insulation layer. The main way to the thermal transfer of the BWR lattice was thermal radiation. The integrated regulation of load-bearing and thermal insulation performance can be achieved by designing the core distributions and the width-thickness ratio.

The lattice structure obtained the thermal insulation and resilient performance simultaneously. Hence, the fracture BWR lattice still retains favorable thermal insulation performance. It benefits from the character of the big wight-thickness ratio, which could suppress the broken and buckling of the rod during compressive failure. This work achieves the preservation of material functionality in load-bearing and functional integrated materials during large deformations and fractures. It provides a new way of designing advanced structural and functional integrated materials.

## Figures and Tables

**Table 1:** Parameters of the BWR lattices.

	*k*	Designed Structure	Printed Structure	Truss Diameter /mm	Cell Size /mm	Lattice Size /mm
S-1	6	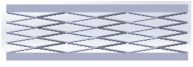	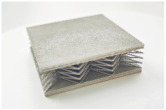	0.75	15 × 15 × 2.5	45 × 45 × 10
S-2	8	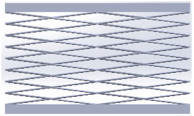	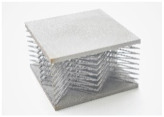	0.75	20 × 20 × 2.5	40 × 40 × 20

**Table 2:** Components of TC4 powders.

Element	Al	V	Fe	O	N	Ti
Component (wt.%)	6.16	4.3	0.163	0.0924	0.0104	Bal.

**Table 3:** Process parameters of 3D printing.

	Scan Strategy	Scan Speed /mm·s^−1^	Laser Power /W	Hatch Spacing /μm	Layer Thickness /μm
Parameters	S type	900	95	60	30

**Table 4:** Parameters of the single core.

	*λ*_0_ /W·m^−1^·°C^−1^	*H* /mm	*L* /mm	*D* /mm	*n*	*m*
S-1	7.96	2.5	15	0.75	9	4
S-2	7.96	2.5	20	0.75	4	8

## Data Availability

The data that support the findings of this study are available from the corresponding author, [X.L.], upon reasonable request.

## Supplementary Materials

The following supporting information can be downloaded at: https://www.mdpi.com/article/10.3390/ma15238625/s1, Figure S1. Magnified image of S-2 after heating.

## References

[1] Han D., Yue K., Cheng L., Yang X., Zhang X. (2020). Measurement of the thermophysical properties of anisotropic insulation materials with consideration of the effect of thermal contact resistance. Materials.

[2] Wu D., Wang Y., Gao Z., Yang J. (2015). Insulation performance of heat-resistant material for high-speed aircraft under thermal environments. J. Mater. Eng. Perform..

[3] Huang Y., Azarmi F., Jazi M.S. Innovative insulations for spacecraft on-surface monitoring system in harsh environments. Proceedings of the Conference on Sensors and Smart Structures Technologies for Civil, Mechanical, and Aerospace Systems, SPIE.

[4] Hou C., Yang G., Wan X., Chen J. (2019). Study of thermo-fluidic characteristics for geometric-anisotropy Kagome truss-cored lattice. Chin. J. Aeronaut..

[5] Chen Z., Jia Z., Yan N. (2017). Effect of insulation core type on thermal conductivity of sandwich structure. J. Compos. Mater..

[6] Wang X., Wei K., Tao Y., Yang X., Zhou H., He R., Fang D. (2019). Thermal protection system integrating graded insulation materials and multilayer ceramic matrix composite cellular sandwich panels. Compos. Struct..

[7] Belardi V.G., Fanelli P., Trupiano S., Vivio F. (2021). Multiscale analysis and mechanical characterization of open-cell foams by simplified FE modeling. Eur. J. Mech.-A Solids.

[8] Mazur M., Leary M., McMillan M., Elambasseril J., Brandt M. (2016). SLM additive manufacture of H13 tool steel with conformal cooling and structural lattices. Rapid Prototyp. J..

[9] Evans A., Hutchinson J., Ashby M. (1998). Multifunctionality of cellular metal systems. Prog. Mater. Sci..

[10] Wicks N., Hutchinson J.W. (2001). Optimal truss plates. Int. J. Solids Struct..

[11] Chen Y., Zhang L., Zhao Y., He R., Ai S., Tang L., Fang D. (2019). Mechanical behaviors of C/SiC pyramidal lattice core sandwich panel under in-plane compression. Compos. Struct..

[12] Zhao M., Zhang D.Z., Liu F., Li Z.H., Ma Z.B., Ren Z.H. (2020). Mechanical and energy absorption characteristics of additively manufactured functionally graded sheet lattice structures with minimal surfaces. Int. J. Mech. Sci..

[13] Liu J., Kanwal H., Tang C., Hao W. (2022). Study on flexural properties of 3D printed lattice-reinforced concrete structures using acoustic emission and digital image correlation. Constr. Build. Mater..

[14] Tao Y., Li P., Zhang H., Shi S.Q., Zhang J., Yin Q. (2022). Compression and flexural properties of rigid polyurethane foam composites reinforced with 3D-printed polylactic acid lattice structures. Compos. Struct..

[15] Xu Y., Xu N., Zhang W., Zhu J. (2019). A multi-layer integrated thermal protection system with C/SiC composite and Ti alloy lattice sandwich. Compos. Struct..

[16] Lv T., Liu M., Zhu D., Gan L., Chen T. (2018). Nanocarbon-Based Materials for Flexible All-Solid-State Supercapacitors. Adv. Mater..

[17] Wang Y., Yang L., Shi X., Shi X., Chen L., Dargusch M., Zou J., Chen Z.-G. (2019). Flexible Thermoelectric Materials and Generators: Challenges and Innovations. Adv. Mater..

[18] Yu Y., Liu F., Liu J. (2017). Direct 3D printing of low melting point alloy via adhesion mechanism. Rapid Prototyp. J..

[19] Guo J., Fu S., Deng Y., Xu X., Laima S., Liu D., Zhang P., Zhou J., Zhao H., Yu H. (2022). Hypocrystalline ceramic aerogels for thermal insulation at extreme conditions. Nature.

[20] Xiao L., Song W. (2018). Additively-manufactured functionally graded Ti-6Al-4V lattice structures with high strength under static and dynamic loading: Experiments. Int. J. Impact Eng..

[21] Liang D., He G., Chen W., Chen Y., Chyu M.K. (2022). Fluid flow and heat transfer performance for micro-lattice structures fabricated by Selective Laser Melting. Int. J. Therm. Sci..

[22] Bai X., Zheng Z., Nakayama A. (2019). Heat transfer performance analysis on lattice core sandwich panel structures. Int. J. Heat Mass Transf..

[23] Yang M., Tao W. (2006). Heat Transfer.

[24] Caogen Y., Hongjun L., Zhonghua J., Xinchao J., Yan L., Haigang L. (2008). A study on metallic thermal protection system panel for Reusable Launch Vehicle. Acta Astronaut..

[25] Wei K., He R., Cheng X., Pei Y., Zhang R., Fang D. (2015). Fabrication and heat transfer characteristics of C/SiC pyramidal core lattice sandwich panel. Appl. Therm. Eng..

[26] De Arrieta I.G., González-Fernández L., Risueño E., Echániz T., Tello M. (2020). Isothermal oxidation kinetics of nitrided Ti-6Al-4V studied by infrared emissivity. Corros. Sci..

[27] Gao G., Li Y., Hu D., Wu Z., Li C., Li Z., Xi Z. (2018). Effect of voltage on infrared emissivity of MAO coatings on TC4 titanium alloys. Titan. Ind. Prog..

