# Supplementary figures and images for: Thermal Insulation and Compressive Performances of 3D Printing Flexible Load-Bearing and Thermal Insulation Integrated Lattice
Source: Materials (Basel). 2022 Dec 2;15(23):8625. doi: 10.3390/ma15238625 (PMC9736691; doi:10.3390/ma15238625)

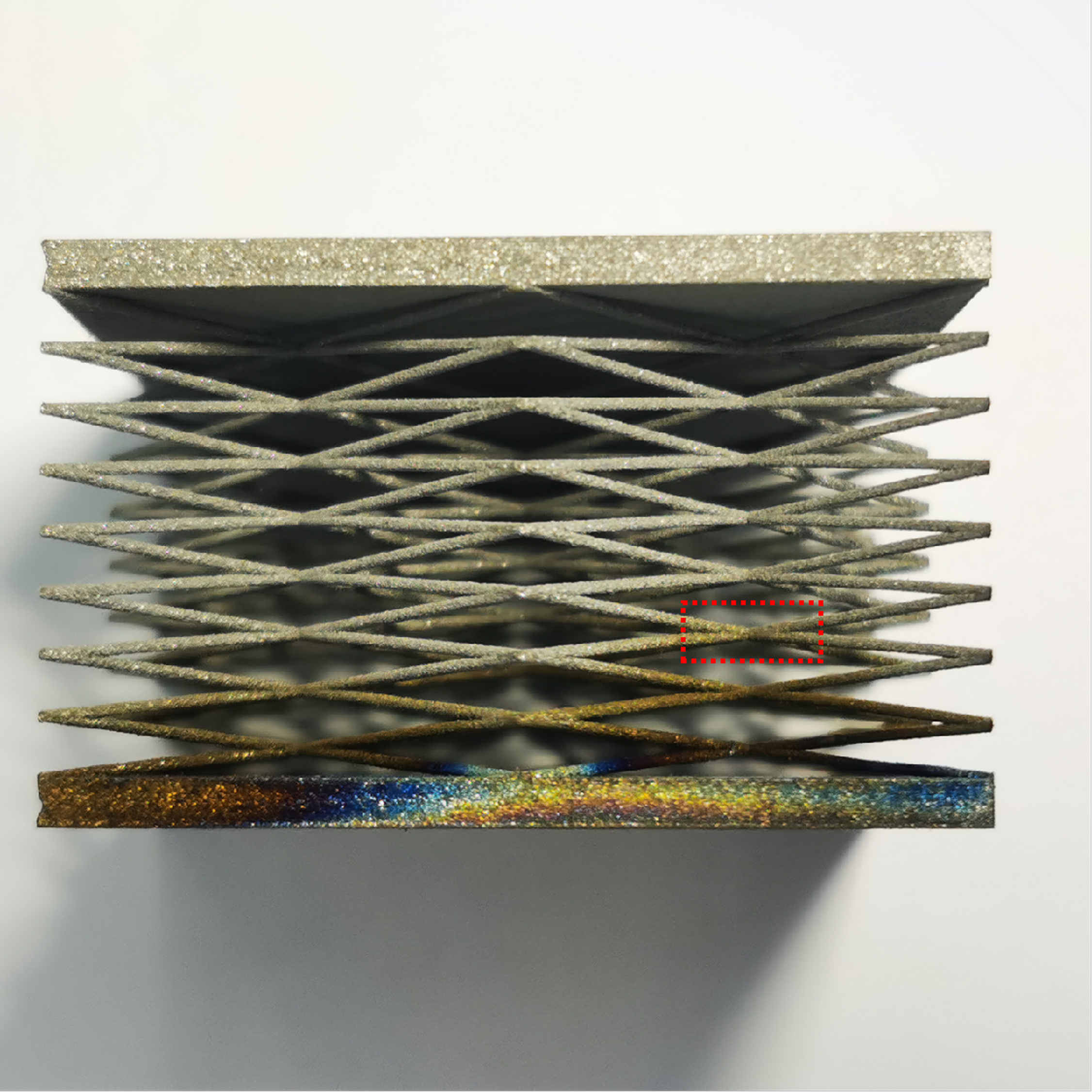

Supplement: Supplementary file 1 [file materials-15-08625-s001.zip › materials-2018795-supplementary.tif]
